# To Medical Microscopists

**Published:** 1889-06

**Authors:** 


### TO MEDICAL MICROSCOPISTS.

In behalf of "THE AMERICAN ASSOCIATION FOR THE STUDY AND CURE OF INEBRIETY" the sum of One Hundred Dollars is offered by Dr. L. D. MASON, Vice-President of the Society, for the best original essay on "*The Pathological Lesions of Chronic Alcoholism, Capable of Microscopic Demonstration.*"

The essay is to be accompanied by carefully prepared microscopic slides, which are to demonstrate clearly and satisfactorily the pathological conditions which the essay considers.

Conclusions resulting from experiments on animals will be admissible. Accurate drawings or micro-photographs of the slides are desired.

The essay, microscopic slides, drawings or micro-photographs, are to be marked with a private motto or legend and sent to the Chairman of the Committee on or before October 1st, 1890.

The object of the essay will be to demonstrate: *First*, Are there pathological lesions due to chronic alcoholism? *Secondly*, Are these lesions peculiar or not to chronic alcoholism?

The microscopic specimens should be accompanied by an authentic alcoholic history, and other complications, as syphilis, should be excluded.

The successful author will be promptly notified of his success, and asked to read and demonstrate his essay personally or by proxy, at a regular or special meeting of the "MEDICAL MICROSCOPICAL SOCIETY" of Brooklyn. The essay will then be published in the ensuing number of "*The Journal of Inebriety*" (T. D. Crothers, Hartford, Conn.) as the prize essay, and then returned to the author for further publication or such use as he may desire. The following gentlemen have consented to act as a Committee:

*Chairman*—W. H. BATES, M. D., F. R. M. S., Lond., Eng.,
(President Med. Microscopical Soc., Brooklyn.)
175 REMSEN ST., BROOKLYN, N. Y.
JOHN E. WEEKS, M. D.,
43 WEST 18TH STREET, NEW YORK.
RICHMOND LENNOX, M. D.,
164 MONTAGUE ST., BROOKLYN, N. Y.

---

### ITEMS.

NASHVILLE, Tenn., has appropriated $20,000 for a new city hospital.

DR. Edward T. Bruen, of Philadelphia, died last week of pneumonia.

LACTOPEPTINE is already in common use, as the standard remedy for indigestion and all nutrient disorders.

THE Iowa City Board of Health has prohibited the sale of Limbuerger cheese, on the ground that it is dangerous to the public health.

THERE are doctors in a great many of the professions Cedric; but most people only give this title to medical men. Dr. generally expresses the exact state of the patient's relations to his physician.—*Puck.*

THE physician who depends upon the gratitude of his patients for his fee, is like the traveller who waited upon the bank of a river until it would finish flowing that he might cross to the other side.—*Canada Lancet.*

WHEN in a crowded Chicago hotel Dubbleby was put in a room with a howling Anarchist, he called the clerk up at midnight to inform him that he was suffering from inflammatory room-mate-ism.—*San Francisco Examiner.*

DR. WM. A. HAMMOND'S SANITARIUM.—Dr. Hammond has now the largest, finest, and most complete sanitarium in the United States. The large experience of the Dr., and the improved means at command, is a sufficient guarantee that patients under his care will be in excellent hands.

A CITIZEN of Iona, Mich., while standing with wet rubbers on an iron door-step, suddenly lost the power of walking. He nearly fainted with terror, thinking he was paralyzed. Upon discovering that his rubbers were frozen to the door-step he felt better.—*N. Y. Med. Times.*

FELLOWS' HYPOPHOSPHITES as prepared by James I. Fellows, Chemist, 48 Vesey St. N. Y., has sustained a high reputation in America and England for efficiency in the treatment of Pulmonary Tuberculosis, and other affections of the respiratory organs. It is also employed in various nervous and debilitating diseases with success. Circulars sent to Physicians on application.

THE CENTURY for the present month, a Centennial number, is devoted mainly to topics suggested by the inauguration of President Washington. The illustrations are rich and numerous; many are copies of unpublished prints and portraits in the possession of private families and private collections. Mr. John Bach McMaster, in "A Century of Constitutional Interpretation," traces, with a masterly hand, the origin of many political problems.

MR. JAGGS HAD THE PROOF.—"I see," observed Mr. Snaggs, "that some eminent men think the Garden of Eden was located in the Mississippi Valley."

"That may be true," replied Mr. Jaggs, "for the ark rested in the Southern States."

"It did?"

"Yes; Noah came out of the Arkansaw land, you know."—*Pittsburgh Chronicle.*

WHAT THE DOCTORS HAVE DONE FOR US.—We are not often permitted to print so admirable a paper as the address of Dr. J. Scott Todd, which will be found elsewhere this morning.

The revelations he makes of the work done by medicine and practice, the lengthening of human life and the alleviation of human ills, will give laymen a new and higher idea of the effectiveness of the profession he so ably represents. It is a comprehensive paper, and bears the mark of such painstaking care that every truth it declares will be accepted, though many of them are startling. Dr. Todd has done his profession a marked service, and the State at large will profit by it.—*Constitution.*

ARCHIVES OF PEDIATRICS.—The April number of the *Archives of Pediatrics* comes to us this month increased to eighty pages, and is especially interesting and attractive. It contains besides the regular monthly contributions by Jacobi on the "Therapeutics of Infancy and Childhood" and Forchheimer on the "Medical

Diseases of the Mouth," either one of which is worth the year's subscription. Townsend on "Acute Lobar Pneumonia in Children," Seibert on "Stomach Washing of Infants," an interesting article (illustrated) on the latest procedure in the treatment of Gastro-intestinal Catarrh, Baruch on the "Treatment of Incontinence of Urine," Earle on "Diphtheria in Chicago," Keating on the "Differential Diagnosis in the Fevers of Childhood," and a large number of brief, practical abstracts from the German, French, and English medical journals of the day.

CALIFORNIA HUMOR: *Delayed Menstruation from an Unusual Cause.*—A druggist in this city was recently consulted by a lady in a case of delayed menstruation, she having passed the regular period by three weeks. She was unable to assign any reason for this unusual occurrence, as she had heretofore been exceptionally punctual. Inquiry developed the fact that she was married and living with her husband. The druggist, a family man of experience, stated that the case was beyond his sphere, but that he thought there was a possible explanation for the suppression. This the patient could not understand, she had "always been so regular." In reply to the question, whether as a married woman a reason for her condition was not somewhat obvious, she answered in the negative, but after a few moments' reflection said: "Yes, I was vaccinated about three weeks since; could that have anything to do with it?" The druggist thought that the vaccination had taken, but he did not say so.—*Occidental Medical Journal.*

RAISING THE STANDARD IN THE UNITED STATES.—It is with unfeigned gratification that we notice the determined movement which the profession of the various States throughout the neighboring Republic is making towards raising the standard of medical education. Knowing as we do the high attainments of many of their teachers, and the thorough course of instruction given by many of their universities, we have always felt sorry to hear the M. D. of American graduates spoken of with contempt by the English and European profession. And yet, how could it be otherwise ? Twelve years ago Buchanan was selling his Philadelphia diplomas of M. D., C. M., for five pounds apiece, and he had been doing this for five years without let or hindrance. It will take thousands of first-class graduates to undo the harm which each of those bogus ones did.—*Ex.*

AGE AND VITALITY.—In a meeting of the Hungarian Academy of Sciences, Joseph Korosi read a paper on "The Influence of Parents' Ages on the Vitality of Children." Mr. Korosi has collected about 30,000 data, and has come to the following conclusions: Mothers under twenty years of age and fathers under twenty-four have children more weakly than parents of riper age. Their children are more subject to pulmonary diseases. The healthiest children are those whose fathers are from twenty-five to forty years of age, and whose mothers are from twenty to thirty years old. Mr. Korosi says that the best marriages are those in which the husband is senior to the wife, but a woman from thirty to thirty-five years old will have healthier children if her husband be somewhat younger than herself. A man from thirty to forty years old ought to take a wife from twenty to thirty. If the mother be five years older than the father the vitality of the children becomes impaired.—*Ex.*

THE ALLEGED ANÆSTHETIC EFFECT OF ARTIFICIAL LOCAL ANÆMIA.—The increased anæsthetic powers of such alkaloids as cocaine and erythrophlœine when injected into a part that has been rendered bloodless, by means of Esmarch's bandage or some other contrivance to accomplish the same end, have been said by some to be more apparent than real, the anæsthesia being alleged to be really due to the anæmia alone. To settle the point, Karewski, of Berlin, ("Therapeutische Monatshefte;" "Deutsche Medizinal-Zeitung") has tested the sensibility of parts that have simply been emptied of their blood, without being subjected to the action of any drug. He finds that certain paræsthesiæ are caused, but that the sensibility to heat and cold is unimpaired, that the tactile perception is sufficient to enable the subject to distinguish the contact of a blunt object from that of a pointed one, and that the sensitiveness to painful impressions, far from being suspended, is rather intensified. Anæmia, then, is not an anæsthetic.—*Ex.*

NEW ANTIDOTE FOR MORPHINE.—Prof. Bokai, of Klausenburg, believes that the best antidote for morphine is picrotoxin. The two substances act in an opposite manner on the respiratory center, morphine paralyzing its action, while small doses of picrotoxin increase it. As in poisoning by morphine, death occurs from paralysis of the respiratory centre, and as picrotoxin hinders this paralysis, it follows that picrotoxin is likely to be of real use in morphine poisoning. In morphine poisoning, diminution of the blood pressure plays an important part, but picrotoxin enjoys the property of stimulating the vasoconstrictor center of the medulla and thus counteracts the effect of the morphine. Once again, the action of these two substances on the cerebral hemispheres is also of an opposite character. As atropine, the only known antidote of morphine, cannot be administered in large doses, it is certainly desirable that no other means of combating morphine poisoning should be sought for. Prof. Bokai thinks that picrotoxin may be useful as a substitute for preparations of nux vomica, and he also believes that it will be found of value in preventing chloroform asphyxia.—*London Lancet.*

THE PREVENTION OF SHOCK BY ATROPINE.—The President also recommended the hypodermatic injection of from 1–75 to 1–100 of a grain of atropine previous to giving ether for an operation. In the second of the two foregoing cases the woman had been etherized, so as to admit of a brief examination a few days before the operation, and had come out of the anæsthetic in a deplorable condition, from which she had not rallied for several hours. For considerable periods the pulse had been very weak and even imperceptible. On the contrary, atropine having been given before the ether on the day of the operation, she was taken from the table with a pulse as good as before the operation began. The subsequent symptoms of shock had been very slight. He had used atropine for this purpose for five or six years, having first observed its control of the inhibitory action on the heart in certain physiological experiments. How much influence it exerted it was not always easy to determine, but he believed it to be of great value.—Dr. L. A. Stimsom in *N. Y. Surgical Society.*

PROFESSIONAL etiquette in England seems to involve in its toils the doctor's wife as well as the doctor himself. A correspondent of the *British Medical Journal* says that his wife, at the request of mutual friends, called upon some new residents in her neighborhood and established a pleasant acquaintance with them. A fellow-practitioner objects to the call, on the ground that the wife represents her husband, and is guilty of a breach of medical etiquette in calling upon people other than her husband's patients. The editor of the *Journal* quotes in reply from the "Code of Medical Ethics:" "Closely akin to solicitation is that of calling upon new residents in the neighborhood and leaving their card, ostensibly as a remark of respect, but in reality to seek for practice. It cannot, therefore, be too deeply impressed upon such that the true dignified practice, and the most consistent with a due respect for self and the faculty, is to wait until their professional or social acquaintance is sought; in such case, moreover, it is far more likely to be appreciated." If the English doctor's wife is willing to submit to an absurd regulation which limits her society to the families among which her husband practices, she must have very little spirit of independence, and bear but a faint resemblance to her sister on this side of the water.—*North West Lancet.*

SOME POPULAR MEDICAL SUPERSTITIONS.—In a book entitled "A Bird's-eye View of France in the Middle Ages," M. Challemel refers to a number of superstitions which were current at that time, many of which have not yet died out. There were several means of warding off fevers. One was to eat neither meat nor eggs at Easter and on other solemn festivals; another to carry about on the person a piece of a human bone; and still another to pluck and eat the first daisy found in the field. In order to cure a fever the sufferer would rise early in the morning, go out into the field, walking backward all the time, pluck a handful of herbs, and without looking at it, throw it behind him, and then return quickly to the house. The fever then forsook him and fastened itself upon the devil. The Bretons preserved their children from all evils by putting on them a damp shirt. A knife with a white handle was a sure preservative against colic. The toothache was quickly relieved by touching the painful part with a dead man's tooth. Running here and there, without particular aim, through a church, was sufficient to ward off pleurisy. The formation of gall-stones was rendered impossible by rolling one's self naked in a field of oats. Spitting in the mouth of a live frog was a very efficacious remedy for a cough. Earache was cured by touching the ear with the hand of a skeleton, and headache was quickly relieved by binding the temples with a cord by which some one had been hung.—*Journal de Médicine et de Chirurgie Pratiques, October, 1888.*

THE "Extra Edition" of the *Journal of the American Medical Association* has come to us full of interest and information. In a special article by Dr. Wm. G. Eggleston of Chicago, it gives a tabulated list of the various medical colleges in the United States, their date of organization, matriculation and graduation requirements, etc., and adds facts which deserve the notice and careful consideration not only of practitioners but students of medicine, and those contemplating entering this field of professional life. Much is being said upon the subject herein treated, the laudable intent of which is to elevate the standard of medical education throughout our country, and we add our voice to the movement, hoping that some steps may be adopted which may at once check the increasing tendency of many little competent and poorly prepared to undertake a work of so great importance and responsibility as the practice of medicine, and aid our colleges, while denying none worthy, in sending out only graduates well equipped for the sacred duties of physicians.

The *Journal* announces the annual meeting of the American Medical Association to be held at Newport, R. I., June 25, 26, 27 and 28, giving a programme of the same, and adds attractive views illustrating this beautiful city by the sea. We join in wishing that the convention may be as pleasant and profitable as those of the past.

THERE are people who believe anything. For instance, the following report was made with imperturbable gravity at a recent meeting of the Philadelphia Obstetrical Society:

A man was seized with vomiting every morning, two weeks after the last appearance of the menses in his wife, who presented no other appearance of pregnancy. The husband continued to vomit for two months. In her previous pregnancies he had presented the same symptoms.

The author of the report does not explain this curious fact. Several of the members present stated that sympathetic vomiting between married people was not very rare. If this be so, it will be necessary in future to classify the signs of pregnancy as those on the part of the husband and those on the part of the wife, the former ranking as presumptive signs.—*Archives de Cologoie.*

